# Editorial: Quantum electromagnetic photon-mediated communication in neuronal networks

**DOI:** 10.3389/fnsys.2026.1804343

**Published:** 2026-02-25

**Authors:** Marco Cavaglià, Marco Pettini, Travis J. A. Craddock, Aristide Dogariu

**Affiliations:** 1 Department of Mechanical and Aerospace Engineering (DIMEAS), Politecnico di Torino, Turin, Italy; 2 Aix Marseille University, CNRS, CPT, Marseille, France; 3 CNRS Centre de Physique Théorique UMR7332, Marseille, France; 4 Quantum Biology Laboratory, Howard University, Washington, DC, United States; 5 Departments of Biology, and Physics & Astronomy, Waterloo Institute for Nanotechnology, University of Waterloo, Waterloo, ON, Canada; 6 Center for Research and Education in Optics and Lasers (CREOL), The College of Optics and Photonics, University of Central Florida, Orlando, FL, United States

**Keywords:** electromagnetic field (EMF), field based information processing, hybrid bioelectromagnetic computation, neural coherence and global integration, ultraweak photon emission (UPE)

Understanding how information is generated, integrated, and propagated in the nervous system remains one of the central challenges of contemporary neuroscience. While synaptic transmission and action-potential-based signaling have provided an extraordinarily successful framework for describing neural computation, growing experimental and theoretical evidence suggests that this picture may be incomplete when it comes to explaining global integration, state-dependent modulation, and the emergence of coherent cognitive phenomena.

Over the last decades, electromagnetic (EM) fields, ultraweak photon emission (UPE), and field-mediated interactions have increasingly appeared in discussions at the interface of neuroscience, biophysics, and quantum biology. Yet these subjects have often remained fragmented across disciplines, sometimes treated as epiphenomena, sometimes over-interpreted, and frequently lacking a common conceptual language. The present Research Topic was conceived to address this fragmentation by offering a focused, interdisciplinary space in which EM- and photon-mediated mechanisms could be examined rigorously, critically, and without *a priori* commitments.

## Rationale and scope of the Research Topic

The aim of this Research Topic was not to promote a single explanatory framework, but rather to explore whether electromagnetic and photonic processes can play a meaningful role in neuronal communication and information processing, and under what conditions such roles are plausible. Importantly, the Research Topic explicitly welcomed theoretical analyses, experimental studies, and negative or constraining results, recognizing that progress in emerging fields depends as much on delimiting what does not work as on proposing new mechanisms.

Across the contributions collected here, a common thread emerges; EM phenomena in biological systems are neither negligible noise nor do they have universal explanations. Instead, they represent a structured layer of physical interaction that must be evaluated with quantitative care, mechanistic specificity, and epistemic restraint.

## Contributions and conceptual axes

Several complementary perspectives are represented among the accepted articles.

The first contribution by McFadden advances a conceptual and computational framework in which neural information processing is described as a hybrid system, combining conventional digital-like neuronal operations with field-based, analog integration mediated by endogenous EM activity. This work situates consciousness and general intelligence within a physically unified EM field, proposing a Hybrid Digital–Electromagnetic Field (HyDEMF) architecture as a possible route toward artificial systems capable of richer integration and adaptive behavior. Rather than presenting this framework as a finalized model, the article positions it as a testable hypothesis space, emphasizing physical plausibility and architectural constraints.

The second contribution by Nevoit et al. offers a broad, critical overview of biophotonic signaling in the human body and brain. By examining ultraweak photon emission as both a metabolic by-product and a potential informational signal, the authors outline current experimental evidence, unresolved methodological challenges, and future directions necessary to discriminate signaling roles from epiphenomenal emission. This work provides an essential grounding, clarifying where biophoton research stands today and what would be required for it to mature into a mechanistically robust field.

Equally important are contributions that establish boundaries. A rigorous theoretical analysis by Talbi et al. demonstrates that the radical pair mechanism, while successful in explaining magnetic field effects in other biological contexts, cannot account for the reported effects of telecommunication-frequency EM fields on reactive oxygen species. By quantitatively showing that the required coupling parameters would exceed biologically plausible values, this study exemplifies the value of negative results in refining the conceptual landscape and redirecting attention toward alternative mechanisms, including electric-field-mediated interactions.

Finally, an experimental study by Ghaffari et al. investigates UPE under different anesthetic conditions, revealing distinct effects associated with ketamine and thiopental. By linking UPE dynamics to oxidative-nitrosative stress and pharmacologically induced states of consciousness, this work provides an empirical anchor for the broader discussion, illustrating how EM and photonic observables may relate to physiological and neurochemical state changes.

## Emerging insights and convergences

Taken together, these contributions suggest several converging insights. First, EM and photonic phenomena in neural systems are real, measurable, and state dependent. Second, their functional relevance cannot be assumed but must be established through careful modeling, controlled experimentation, and falsifiable predictions. Third, field-mediated processes may be particularly relevant for understanding global integration, modulation, and transitions between functional states, rather than the local point-to-point computation alone.

At the same time, the Research Topic underscores the importance of epistemic discipline. Not every observed electromagnetic or photonic level effect implies information processing, and not every limitation of synaptic models necessitates quantum explanations. The strength of this Research Topic lies precisely in its balanced treatment of possibility and constraint.

## Limitations and open questions

Despite the progress represented here, substantial challenges remain. Direct causal evidence linking electromagnetic fields to specific informational roles in neural computation is still limited. Methodological standardization improved spatiotemporal resolution, and better synergy between theory and experiment are urgently needed. Moreover, translating these insights into artificial systems raises non-trivial engineering questions regarding detectability, controllability, and robustness of field-based information encoding.

## Outlook

Looking forward, research at the intersection of neuroscience, biophysics, and electromagnetics will benefit from integrative approaches that combine negative results, conceptual modeling, and carefully designed experiments. Future work may explore how electric and magnetic field components interact with ion channels, membranes, and intracellular structures, how photonic emission correlates with metabolic and cognitive states, and whether hybrid computational architectures can exploit field-based dynamics without abandoning classical physical principles.

By bringing together diverse yet complementary contributions, this Research Topic aims to provide a coherent snapshot of an evolving field, one that is neither speculative enthusiasm nor conservative dismissal, but a disciplined exploration of the physical substrates of neural information processing.

